# Discriminating Glioblastoma from Peritumoral Tissue by a Nanohole Array-Based Optical and Label-Free Biosensor

**DOI:** 10.3390/bios13060591

**Published:** 2023-05-30

**Authors:** Víctor García-Milán, Alfredo Franco, Margarita Estreya Zvezdanova, Sara Marcos, Rubén Martin-Laez, Fernando Moreno, Carlos Velasquez, José L. Fernandez-Luna

**Affiliations:** 1 Department of Neurological Surgery and Spine Unit, Hospital Universitario Marqués de Valdecilla, 39008 Santander, Spain; victor.garcia@scsalud.es (V.G.-M.); ruben.martin@scsalud.es (R.M.-L.); carlosjose.velasquez@scsalud.es (C.V.); 2 Department of Applied Physics, Faculty of Sciences, Universidad de Cantabria, 39005 Santander, Spain; alfredo.franco@unican.es (A.F.); fernando.moreno@unican.es (F.M.); 3 Instituto de Investigación Marqués de Valdecilla (IDIVAL), 39012 Santander, Spain; estrellanik_zv@hotmail.es; 4 Servicio de Anatomía Patológica, Hospital Universitario Marqués de Valdecilla, 39008 Santander, Spain; sara.marcos@scsalud.es; 5 Department of Anatomy and Cell Biology, Universidad de Cantabria, 39005 Santander, Spain; 6 Genetics Unit, Hospital Universitario Marqués de Valdecilla, 39008 Santander, Spain

**Keywords:** glioblastoma, biosensor, extraordinary optical transmission, refractive index

## Abstract

In glioblastoma (GBM) patients, maximal safe resection remains a challenge today due to its invasiveness and diffuse parenchymal infiltration. In this context, plasmonic biosensors could potentially help to discriminate tumor tissue from peritumoral parenchyma based on differences in their optical properties. A nanostructured gold biosensor was used ex vivo to identify tumor tissue in a prospective series of 35 GBM patients who underwent surgical treatment. For each patient, two paired samples, tumor and peritumoral tissue, were extracted. Then, the imprint left by each sample on the surface of the biosensor was individually analyzed, obtaining the difference between their refractive indices. The tumor and non-tumor origins of each tissue were assessed by histopathological analysis. The refractive index (RI) values obtained by analyzing the imprint of the tissue were significantly lower (*p* = 0.0047) in the peritumoral samples (1.341, Interquartile Range (IQR) 1.339–1.349) compared with the tumor samples (1.350, IQR 1.344–1.363). The ROC (receiver operating characteristic) curve showed the capacity of the biosensor to discriminate between both tissues (area under the curve, 0.8779, *p* < 0.0001). The Youden index provided an optimal RI cut-off point of 0.003. The sensitivity and specificity of the biosensor were 81% and 80%, respectively. Overall, the plasmonic-based nanostructured biosensor is a label-free system with the potential to be used for real-time intraoperative discrimination between tumor and peritumoral tissue in patients with GBM.

## 1. Introduction

Glioblastoma (GBM) is the most common and lethal of primary brain tumors in adults, accounting for 49.1% of all malignant tumors with an incidence rate of 3.23 cases per 100,000 inhabitants in the United States [1]. Presently, GBM remains a major clinical challenge with a median survival of 14.6 months despite the current standard of care based on surgical resection followed by temozolomide, an alkylating agent, and radiotherapy [2].

The current paradigm of surgical treatment is to achieve maximal safe tumor resection, and the extent of resection is considered a prognostic factor for longer survival [3,4]. Nevertheless, due to GBM’s invasiveness and its diffuse parenchymal infiltration, complete tumor resection is often precluded [5]. Moreover, this infiltrative behavior results in a blurred tumor margin, which hampers the intraoperative identification of the tumor edge by the neurosurgeon [6].

In this context, several tools have been developed attempting to discriminate tumors from the surrounding parenchyma, such as neuronavigation, fluorescent marking, histopathological analysis, and confocal laser endomicroscopy (CLE) [7,8,9,10,11]. However, reliable identification of tumor margins in real time to attempt complete tumor removal remains a challenge today.

Biomedical applications of the extraordinary optical transmission (EOT) phenomenon [12] to discriminate different molecules and tissues have been widely explored in the last few decades [13,14,15]. The EOT phenomenon is usually observed in metal films with periodic nanohole arrays, and it is highly dependent on the changes in the optical properties of the medium in contact with the metal surface due to changes in plasmonic resonance conditions. Different plasmonic-based biosensor systems have been developed to detect very small amounts of biomolecules, taking advantage of the electric field enhancement in plasmonic materials [15,16,17]. Plasmonic biosensors have been proposed for the analysis of clinically relevant biomarkers, such as amyloid beta protein in Alzheimer’s disease [18] or prostate-specific antigen (PSA) in prostate cancer [19]. Moreover, EOT-based technology opens the possibility of using portable diagnostic devices made with miniaturized biosensors without the need for additional sophisticated equipment or specialized personnel to operate them [14,20].

Our group has previously reported the development of an optical system able to perform label-free, highly sensitive, and specific discrimination between live single tumor cells and normal cells in contact with a gold nanohole array [21,22,23]. The performance of the system was validated through the analysis of more than one thousand live cells [21]. We also described the use of the biosensor to study key features of cancer cells [24].

In the present study, we report the performance of the EOT-based biosensor for ex vivo discrimination between tumor and peritumoral tissue in patients with GBM, which provides proof of concept for its potential use as an intraoperative biosensing device.

## 2. Materials and Methods

### 2.1. Design and Subjects

A prospective study was performed, including a series of patients with GBM who were candidates for surgical treatment. Patients met all the following inclusion criteria: (1) adults ≥ 18 years of age; (2) newly diagnosed GBM; and (3) histopathology-confirmed GBM.

The collection of tissue specimens did not modify the standard of care for the patient. Patients’ data were treated confidentially, and research was conducted in accordance with the ethical standards of the Helsinki Declaration.

### 2.2. Clinical and Radiological Variables

Clinical and radiological data were collected and included age, sex, aim of surgery, tumor location, and presence of residual tumor following the surgical procedure.

Moreover, preoperative planification was determined in the preoperative magnetic resonance imaging (MRI) by a semi-automatic manual segmentation process using the Brainlab Elements software modules, Cranial 3.0 (Brainlab AG, Munich, Germany). The tumor area was defined as the contrast-enhanced tumor portion in the contrast-enhanced T1-weighted sequence (CE-T1W), and the peritumoral area as the peritumoral T2/FLAIR hyperintensity in the 3D FLAIR sequence.

### 2.3. Specimens Collection

Tumor and paired adjacent peritumoral tissue specimens were obtained from each case following the routine surgical sampling protocol for histopathological diagnosis. Specimens were collected by the neurosurgeon in the operating room during a standard craniotomy for tumor removal/biopsy. Surgery was performed in a standard manner, following our common institutional practice and the standard of care.

Both specimens were obtained by using MRI-based neuronavigation guidance and intraoperative fluorescence with 5-ALA or sodium fluorescein. The tumor specimen was collected from the tumor core, which was defined as the contrast enhancement T1 hyperintensity in the preoperative MRI. Areas of obvious necrosis and areas with high blood content were avoided. Comparably, the peritumoral sample was taken from the peritumoral normal-appearing parenchyma immediately adjacent to the tumor, avoiding areas of the cortex.

**Figure 1:**
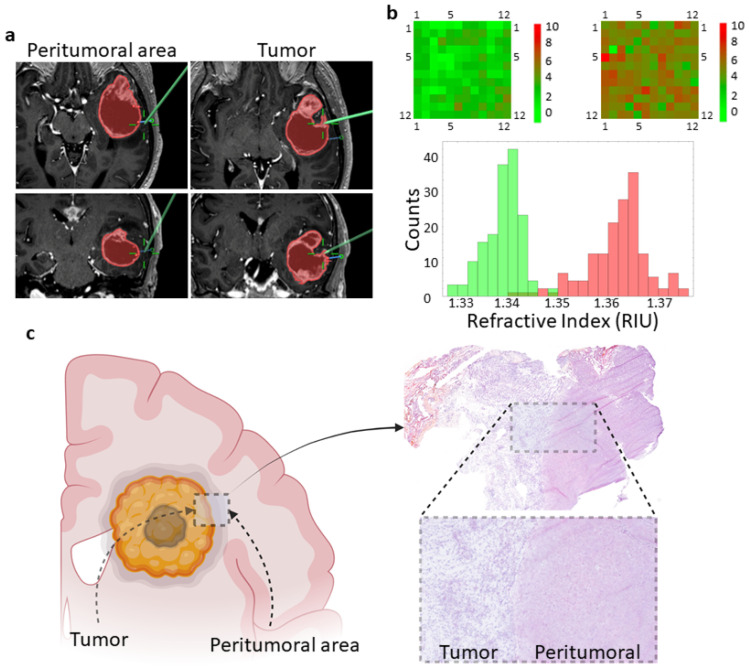
Overview of the workflow. (**a**) Axial (upper panels) and coronal (lower panels) MRI images showing the tumor area (red) of a patient with a left temporal lobe GBM. The tumor area is defined by the contrast-enhanced portion of the T1-weighted sequence and is preplanned in the Brainlab Elements software. Green lines pinpoint the specific sites where the tissue samples were taken. (**b**) Heatmap showing the shift of the plasmon resonance wavelength at each position on the biosensor due to the RI of the imprint. A total of 144 measurements covering the entire biosensor were obtained for each tissue sample. A representative histogram showing the RI values of the imprints left by peritumoral (green) and tumor (red) tissues is shown. RIU, refractive index units. (**c**) Schematic representation of tumor and peritumoral areas of a GBM showing the tumor border and the necrotic tumor core. The right image shows a representative hematoxylin-eosin-stained section revealing the tumor-peritumoral tissue border.

In all cases, the coordinates of the sample collection sites were saved in the navigation system, and screenshots were acquired to determine the precise site of the sampling (Figure 1a). The size of each sample was within a range of 5–8 mm.

### 2.4. Biosensor Optimization and Specimen Processing

The features of the biosensor have already been described in detail [21]. In brief, it consists of a gold film with a square array of holes periodically distributed along an area of 500 m × 500 m made by electron beam lithography (EBL). The hole’s diameter is 220 nm, the array periodicity is 550 nm, and the film thickness is 60 nm. All biosensors were characterized and cleaned before use. Characterization was carried out to determine their optical sensitivity in nm/RIU units and to calibrate the response of each biosensor along the entire surface, as described elsewhere [23]. In order to remove organic residues, biosensors were exposed to piranha solution and plasma cleaning procedures, as reported [21].

Each tumor and peritumoral tissue sample was taken with tweezers and placed on the corresponding biosensor to cover the entire nanohole array. This process has been integrated as part of the routine histopathological examination protocol following standards applied to GBM surgery. In order to get an imprint of the tissue, samples remained in contact with the biosensor surface for 1 min. At the end of this time, the tissue was removed, placed in a properly identified formalin-containing tube, and the histology was assessed by an experienced pathologist (S.M.). The excess liquid on the biosensors was evaporated at room temperature for 5–10 min, and the biosensors were stored at 4 °C until optical analysis.

### 2.5. Optical System and Setup

The imprint left by the tissue on the biosensor was analyzed by means of the optical transmission spectral shift due to the refractive index (RI) of the imprint. The spectral measurements were carried out in regions restricted to a diameter of 40 m, in such a way that each imprint was analyzed 144 times for statistical significance (Figure 1b). 

**Figure 2:**
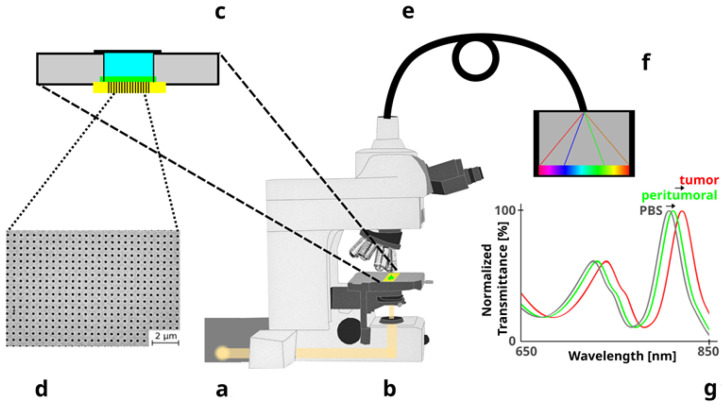
Main parts of the biosensor device. (**a**) Halogen lamp as source of light. (**b**) Adapted upright microscope. (**c**) Sample holder with the biosensor (in yellow with vertical lines) at the bottom of a well that is filled with saline buffer during the measurements. (**d**) Scanning electron micrograph of the nanostructured gold film. (**e**) Optical fiber. (**f**) Spectrophotometer. (**g**) Representative spectral shift due to optical differences between GBM tumors and peritumoral tissues. As a background reference, the spectrum of phosphate buffered saline (PBS) is also included.

The optical system used for the measurements has already been described in detail [21,23]. Briefly, it is composed of a motorized Nikon Eclipse microscope with a 20× objective, an Andor Shamrock 500i spectrograph, and a cooled Idus CCD camera coupled to the spectrograph. The transmission spectra were processed and analyzed using in-house software. A scheme of the optical system is represented in Figure 2.

### 2.6. Pathological Anatomy

After the imprint process, tissue samples were formalin-fixed and paraffin-embedded for histopathological analysis. In brief, 3 μm sections were obtained, and hematoxylin-eosin staining was performed according to standard protocols. Sections were examined under a conventional optical microscope (Figure 1c). Histological diagnosis was completed according to the 2021 WHO classification [25].

### 2.7. Statistical Analysis

A descriptive analysis of each clinical and radiological variable was performed. The Kolmogorov-Smirnov test was used to study the distribution of each variable. Medians and interquartile ranges (IQR) were calculated for variables with a non-normal distribution, while those with a normal distribution were recorded as the mean and standard deviation (SD). 

A Wilcoxon-Mann-Whitney test (WMW) was used to compare non-normal distributed quantitative variables between tumor and peritumoral samples. Statistical significance was defined as a two-tailed *p*-value less than 5% (*p* < 0.05). The statistical analysis was performed using GraphPad Prism version 9.0.0 for Mac OS X (GraphPad Software, San Diego, CA, USA).

The receiver operating characteristic (ROC) curve was used to analyze the critical values to differentiate tumor from peritumoral tissue, and sensitivity and specificity values were obtained. Finally, thresholds for RI were set following the Youden index optimization criteria [26].

## 3. Results

### 3.1. Demographics

A total of 35 GBM patients who underwent surgery at our institution from May 2019 to March 2023 were included in the study. Four cases were excluded due to technical problems with the biosensor and/or the optical measurements. The main clinical, radiological, and demographic features are summarized in Table 1. 

A total of 31 paired tumor-peritumoral samples were analyzed by an expert pathologist, and all tumor samples were histopathologically confirmed GBM (grade IV, WHO 2021) [25].

### 3.2. Biosensor Performance

**Figure 3:**
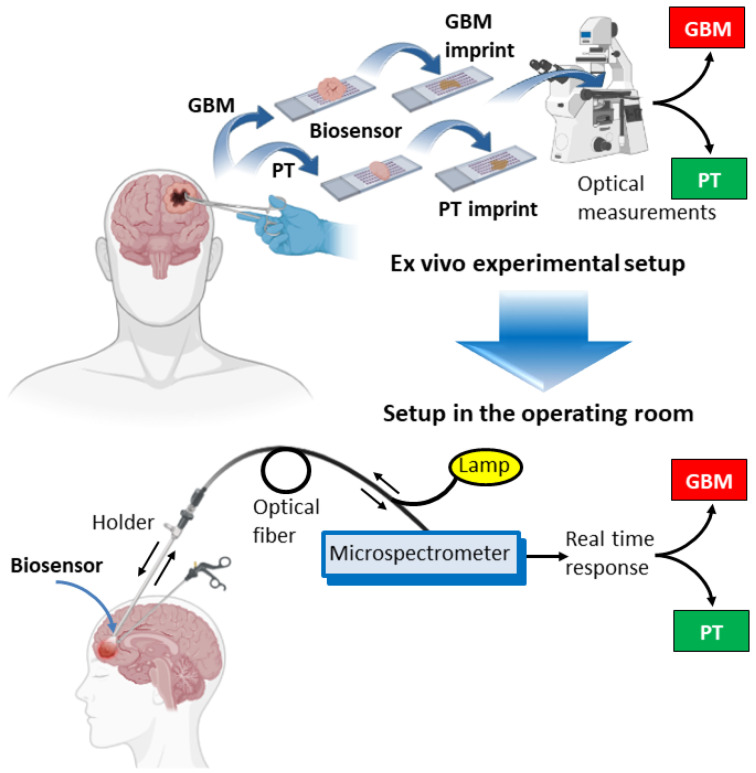
Representation of the experimental system and the proposed setup in the operating room. The upper panel shows the different steps, starting with tissue collection, which is deposited onto the biosensor. Then, tissue is removed, and optical measurements of the imprints left by both GBM and peritumoral tissues are obtained. The lower panel shows a representation of the proposed setup that would be used during a surgical procedure. Light passes through the fiber towards the biosensor, located at the tip of a holder, and is reflected back to a microspectrometer for real-time identification of the tissue. Arrows indicate the direction of the light. Illustration adapted from templates by BioRender.com.

A schematic representation of the entire process is shown in Figure 3, including the proposed adaptation of the ex vivo biosensor system to a setup in the operating room. 

Tissue samples were obtained using neuronavigation guidance and immediately deposited on the biosensor. The RI of tissue imprints was then analyzed for both tumor and peritumoral samples (Table 2). In some cases, postoperative histopathologic examination revealed that the peritumoral tissue was GBM. The RI difference is considered positive when tumor RI is higher than peritumoral RI and negative when peritumoral RI is higher than or very similar to tumor RI. Mean RI values were 1.341 (IQR 1.339–1.349) for peritumoral imprint and 1.350 (IQR 1.344–1.363) for tumor imprint (*p* = 0.0047).

**Figure 4:**
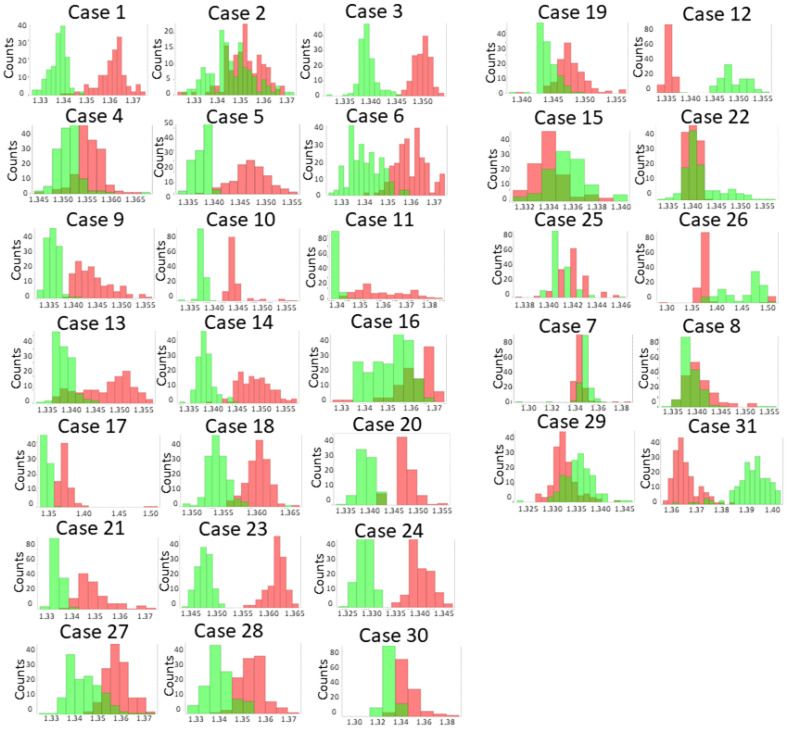
Optical measurements of tissue imprints. Histograms show the RI values of the imprints left on the biosensor by peritumoral (green) and tumor (red) tissue. A magnified image of a representative histogram is shown in Figure 1B.

The RI difference was positive in 22 of the 31 cases (71%), confirming the capacity of the biosensor to distinguish between the tumor and the surrounding peritumoral tissue (Figure 4). The histopathological analysis revealed that in case 19, the peritumoral sample was GBM, indicating a false-positive result of the biosensor.

In cases 7, 8, 29, and 31, the RI difference was negative despite the fact that both tissues were GBM based on the histological assignment. In the other 5 cases, 12, 15, 22, 25, and 26, a negative RI difference was also obtained, but this time the histology confirmed the tumor and peritumoral origin of the tissues. A likely explanation for these unexpected results could be contamination with red blood cells, as they have a high RI (1.370–1.420) [27]. Although areas with high blood content were avoided, the presence of limited numbers of red cells might interfere with optical measurements.

**Figure 5:**
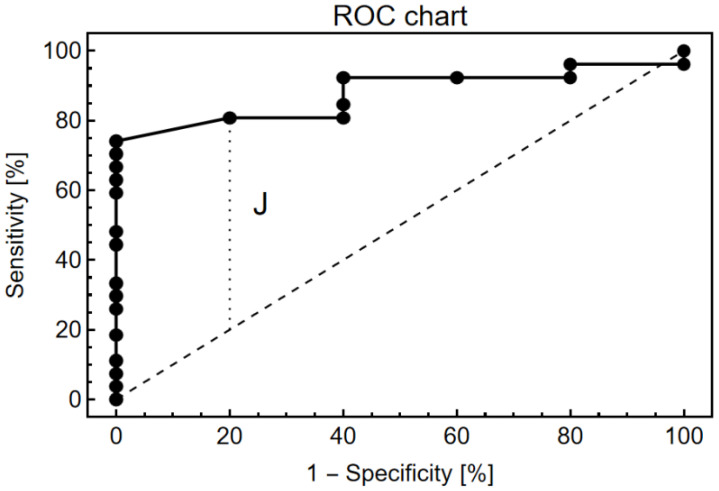
Receiver operating characteristic plot showing the biosensor’s capacity to discriminate between tumor and peritumoral tissues.

The receiver operating characteristic (ROC) curve was used to assess the sensitivity and specificity for each of the possible cut-off points for the discrimination of tumor and peritumoral tissue (Figure 5). The highest Youden index, labeled J in the Receiver Operating Characteristic (ROC) curve, was used to provide an optimal RI cut-off point of 0.003. Under these conditions, the sensitivity and specificity were 81% and 80%, respectively. The area under the curve was 0.8779 (0.7571–0.9988, 95% confidence interval, *p* < 0.0001), indicating that the biosensor is a suitable system to distinguish GBM from its surrounding tissue.

Moreover, the variation of spectral measurements from a single sample was around 0.151 nm, which gives an uncertainty of less than 0.001 RIU.

## 4. Discussion

### 4.1. Discrimination Capacity of the Biosensor

In this study, we prospectively evaluated the accuracy of an EOT-based tool in providing ex-vivo intraoperative discrimination between the GBM tumor and the peritumoral tissue. Other technologies are working on near-real-time assessment for tumor discrimination in GBM [11]. However, this is the first study on the potential use of EOT-based systems as intraoperative biosensing devices for real-time detection of GBM tumor margins during the surgical act.

Interestingly, our optical system was able to discriminate between GBM and peritumoral tissue without any need for tissue preparation or labeling, raising the possibility of implementing such technology during tumor removal in the operating room. 

The ROC curve analysis observed in Figure 5 shows the sensitivity and specificity of each of the possible cut-off points of the EOT-based biosensor. In order to determine the optimal cut-off point to discriminate tumors from peritumoral tissue, the highest Youden index (0.61) was used. The biosensor showed a sensitivity of 81% and a specificity of 80%. A high sensitivity of the system is interpreted as the probability of obtaining a positive result for those tissues that are truly GBM, which allows discrimination from the surrounding parenchymal tissue. On the other hand, high specificity consists of the probability that in a non-tumor tissue, the biosensor will detect RI values corresponding to the peritumoral area.

The data presented here support the application of the nanoplasmonic biosensor to differentiate tumor tissue during tumor resection and may entail several potential advantages over the systems currently being used for the same purpose.

Histopathological analysis is the current gold standard for the identification of tumor tissue, but its definitive result is only provided days after surgery [28]. Intraoperative pathological assessment of tumor margins may guide neurosurgeon intervention, but it does not provide real-time results and requires interpretation by an experienced neuropathologist [29,30]. On the contrary, the nanoplasmonic biosensor can efficiently differentiate between tumor tissue and the surrounding parenchyma and could be used as a real-time discrimination system in GBM surgeries. The system would not require interpretation or special training, as the biosensor could provide a different signal depending on the tissue analyzed.

Other intraoperative techniques used to discriminate tumor tissue, such as fluorescence-guided surgery and confocal laser endomicroscopy (CLE), rely on the pre- or intraoperative administration of a fluorophore to enhance the tumor tissue [9,10,11]. Intraoperative fluorophores commonly used in GBM surgery include 5-aminolevulinic acid (5-ALA) [9,31] and sodium fluorescein [32,33], which are administered to the patient before or during surgery. Although it has been demonstrated that tumor fluorescence derived from 5-ALA enables more complete resections, achieving a progression-free survival improvement [9], fluorescence fades away from the tumor core, and its interpretation becomes challenging at the infiltrative margins [5]. Some authors described key factors that limit the sensitivity of this approach, like low spatial resolution due to the “averaging effect” or poor optical detection for disseminated and sparse cells in tumor margins [31]. Additionally, although 5-ALA and sodium fluorescence have been described as well-tolerated compounds with a low rate of side effects in high-grade glioma resection surgery [34], some studies have described mild side effects, such as intraoperative hypotension in a small proportion of patients [35,36], photosensitivity, and allergic reactions.

Similarly, CLE is a novel labeling technique that allows in vivo digital biopsies for the visualization of cellular structures by using previously injected sodium fluorescein [11]. Recent studies have shown that CLE is a useful tool for the accurate diagnosis of brain tumor lesions by obtaining high-quality intraoperative images of different targets (tumor and peritumoral areas or the interface between both) [37,38]. However, CLE images require interpretation by experts in the field. In addition, fluorescence-based techniques may have side effects, and pregnant patients or patients with hypersensitivity to the fluorophore or renal failure are excluded [38].

On the other hand, the use of the plasmonic biosensor in this context is a label-free method that can accurately discriminate GBM from the peritumoral tissue based on the EOT, which relies on the biophysical features of the tissue. Contrary to fluorescence-guided surgery and the CLE, the plasmonic biosensor would not require any procedure to prepare the patient for surgery, avoiding possible alterations of the tissue as well as potential side effects. In addition, the biosensor does not require interpretation of results because it gives an immediate yes or no response based on a cut-off point for refractive index values.

There are other image-based systems, such as neuronavigation, intraoperative ultrasound (ioUS), and intraoperative MRI (ioMRI). The first one relies on the placement of sensors during surgery that correlate the location with a preoperative neuroimaging study (CT or MRI). However, during surgery, the brain parenchyma undergoes distortion due to cerebrospinal fluid loss, edema, and tumor resection. These changes, known as “brain shift”, make the preoperative neuronavigator image less reliable during surgery [7,8]. ioUS provides real-time feedback, but it has a high user dependency and requires a long learning curve and a complex interpretation of results [39,40]. Lastly, ioMRI can improve the extent of resection by identifying residual tumors during glioma surgery. However, the process of introducing and maintaining iMRI is very costly and time-consuming without real-time feedback [41,42,43], and its use may also increase complications due to prolonged operating time [44,45].

Overall, the nanoplasmonic biosensor capacity for discrimination between tumor and peritumoral tissue, alone or in combination with other methods, may have a place in the workflow for the surgical treatment of GBM.

### 4.2. Limitations and Future Perspectives

Our study included a limited number of patients, and the results will need to be replicated in larger cohorts based on multicenter studies. 

In addition, further optimization of the nanostructure of the plasmonic biosensor could improve performance in the identification of tumor tissue, increasing sensitivity. We realize that other biological components, including red blood cells, may interfere with the RI measurements, but both tissues, GBM and PT, are so close together that we would not expect a contamination bias in a specific tissue. Moreover, tissues were gently flushed with saline buffer once the tissue was deposited on the biosensor, similar to the flushing with saline to remove blood from the surgical field. The sensitivity and specificity of the biosensor are now about 80%. In order to improve specificity, we first need to gain an understanding of the biological basis of the optical differences between tissues, which will reveal the potential contaminants that might interfere with optical measurements.

We are now working on technical issues for a future adaptation of the biosensor to the surgical workflow. Finally, studies are under way to analyze the components in the tumor and peritumoral tissues responsible for the discrimination capacity of the biosensor.

## 5. Conclusions

The plasmonic-based nanostructured biosensor has been shown to efficiently discriminate between tumor and peritumoral tissue ex vivo in patients with GBM. The experimental workflow starts with small pieces of GBM and peritumoral tissue that are deposited on the surface of the biosensor. The imprints left by the tissues are then analyzed with an adapted upright microscope connected to a spectrometer to obtain optical measurements. The sensitivity and specificity of the biosensing system were determined to be about 80%, and the area under the curve was 0.8779 (0.7571–0.9988, 95% confidence interval, *p* < 0.0001), indicating that the biosensor provides a suitable procedure to distinguish GBM from its peritumoral tissue. This is the first step in the road map to transfer the experimental setup to an in vivo system able to discriminate between both tissues in real time, which would assist decision-making during surgery.

To achieve this goal, further optimization of the optical system to improve specificity, an understanding of the biological bases for these optical differences, and studies in larger cohorts of patients undergoing surgery for GBM will need to be done.

## Figures and Tables

**Table 1:** Clinical, sociodemographic, and radiological features.

Case	Age	Sex	Side	Location	Aim of Surgery	Residual Tumor
1	71	Female	Right	Frontal	Resection	No
2	59	Female	Right	Temporal	Biopsy	NA
3	72	Male	Right	Frontal	Biopsy	NA
4	49	Male	Right	Frontal	Resection	No
5	75	Female	Right	Frontal	Resection	Yes
6	69	Male	Right	Temporal	Biopsy	NA
7	50	Male	Left	Frontal	Resection	Yes
8	71	Male	Right	Frontal	Resection	No
9	72	Female	Right	Frontal	Biopsy	Yes
10	71	Female	Right	Parietal	Resection	NA
11	48	Male	Bilateral	Frontal	Biopsy	NA
12	59	Male	Right	Temporal	Resection	Yes
13	58	Female	Right	Temporal	Resection	Yes
14	42	Male	Right	Frontal	Resection	Yes
15	69	Male	Right	Occipital	Resection	No
16	70	Male	Right	Temporal	Resection	No
17	76	Female	Right	Frontal	Biopsy	NA
18	73	Male	Left	Frontal	Resection	No
19	58	Male	Left	Frontal	Biopsy	NA
20	77	Female	Left	Temporal	Resection	Yes
21	66	Male	Left	Occipital	Resection	No
22	68	Male	Left	Frontal	Resection	Yes
23	71	Female	Left	Parietal	Resection	No
24	65	Male	Right	Frontal	Resection	Yes
25	51	Male	Left	Frontal	Resection	No
26	65	Female	Right	Occipital	Resection	Yes
27	68	Male	Left	Temporal	Biopsy	NA
28	49	Male	Right	Frontal	Resection	Yes
29	78	Female	Right	Frontal	Resection	No
30	68	Male	Left	Occipital	Resection	Yes
31	61	Male	Right	Temporal	Resection	Yes

NA, data not available.

**Table 2:** RI values of tumor and peritumoral imprints on the biosensor.

Case	Peritumoral RI	Tumor RI	RI Difference	Peritumoral Histology *	Tumor Histology *
1	1.339	1.362	POS	Peritumoral	GBM
2	1.345	1.351	POS	Peritumoral	GBM
3	1.339	1.350	POS	Peritumoral	GBM
4	1.352	1.356	POS	Peritumoral	GBM
5	1.337	1.349	POS	Peritumoral	GBM
6	1.346	1.365	POS	Peritumoral	GBM
7	1.352	1.348	NEG	GBM	GBM
8	1.342	1.344	NEG	GBM	GBM
9	1.335	1.348	POS	Peritumoral	GBM
10	1.333	1.344	POS	Peritumoral	GBM
11	1.343	1.372	POS	Peritumoral	GBM
12	1.354	1.337	NEG	Peritumoral	GBM
13	1.339	1.350	POS	Peritumoral	GBM
14	1.335	1.344	POS	Peritumoral	GBM
15	1.336	1.334	NEG	Peritumoral	GBM
16	1.353	1.366	POS	Peritumoral	GBM
17	1.340	1.367	POS	Peritumoral	GBM
18	1.356	1.361	POS	Peritumoral	GBM
19	1.346	1.349	POS	GBM	GBM
20	1.341	1.344	POS	Peritumoral	GBM
21	1.349	1.368	POS	Peritumoral	GBM
22	1.345	1.343	NEG	Peritumoral	GBM
23	1.349	1.373	POS	Peritumoral	GBM
24	1.330	1.343	POS	Peritumoral	GBM
25	1.341	1.342	NEG	Peritumoral	GBM
26	1.481	1.399	NEG	Peritumoral	GBM
27	1.341	1.359	POS	Peritumoral	GBM
28	1.344	1.359	POS	Peritumoral	GBM
29	1.342	1.337	NEG	GBM	GBM
30	1.317	1.340	POS	Peritumoral	GBM
31	1.389	1.363	NEG	GBM	GBM

POS, positive; NEG, negative; * Postoperative histopathologic examination.

## Data Availability

All data are included in the article.
